# Tuberculosis Immunity: Opportunities from Studies with Cattle

**DOI:** 10.1155/2011/768542

**Published:** 2010-12-06

**Authors:** W. Ray Waters, Mitchell V. Palmer, Tyler C. Thacker, William C. Davis, Srinand Sreevatsan, Paul Coussens, Kieran G. Meade, Jayne C. Hope, D. Mark Estes

**Affiliations:** ^1^National Animal Disease Center, Agricultural Research Service, US Department of Agriculture, Ames, IA 50010, USA; ^2^Department of Veterinary Microbiology and Pathology, College of Veterinary Medicine, Washington State University, Pullman, WA 99164, USA; ^3^Departments of Veterinary Population Medicine and Veterinary and Biomedical Sciences, University of Minnesota, St Paul, MN 55108, USA; ^4^Molecular Biology and Molecular Virology, Department of Animal Science, Michigan State University, East Lansing, MI 48824, USA; ^5^Animal Bioscience Centre, Teagasc, Grange, BT55&GE Co. Meath, Ireland; ^6^Livestock Diseases Programme, Institute for Animal Health, Compton, Near Newbury RG20 7NN, UK; ^7^Department of Microbiology and Immunology, University of Texas Medical Branch, Galveston, TX 77555-1070, USA

## Abstract

*Mycobacterium tuberculosis* and *M. bovis* share >99% genetic identity and induce similar host responses and disease profiles upon infection. There is a rich history of codiscovery in the development of control measures applicable to both human and bovine tuberculosis (TB) including skin-testing procedures, *M. bovis* BCG vaccination, and interferon-*γ* release assays. The calf TB infection model offers several opportunities to further our understanding of TB immunopathogenesis. Recent observations include correlation of central memory immune responses with TB vaccine efficacy, association of SIRP*α* ^+^ cells in ESAT-6:CFP10-elicited multinucleate giant cell formation, early *γδ* T cell responses to TB, antimycobacterial activity of memory CD4^+^ T cells via granulysin production, association of specific antibody with antigen burden, and suppression of innate immune gene expression in infected animals. Partnerships teaming researchers with veterinary and medical perspectives will continue to provide mutual benefit to TB research in man and animals.

## 1. Introduction: History of Codiscovery

Three essential tools developed in cattle and used for the control of human tuberculosis (TB) include (1) vaccination with an attenuated *Mycobacterium bovis* Bacillus Calmette Guerin (BCG, [1]), (2) use of tuberculin as an *in vivo* diagnostic reagent, and (3) antigen-induced interferon- (IFN-) **γ** as an *in vitro* biomarker of TB infection. In 1913 at the Pasteur Institute (Lille, France), Albert Calmette and Camille Guerin vaccinated 9 cows with *M. bovis* (Nocard strain) attenuated by serial passage on glycerol-soaked potato slices in ox bile (i.e., BCG) [1]. All 9 animals were protected from challenge with virulent *M. bovis,* thereby, demonstrating the potential use of BCG vaccination against *M. tuberculosis* infection of humans. In 1921, BCG was administered to a newborn child (6 mg orally) and has since been used widely for the control of human TB. Within a few years of the discovery of tuberculin by Robert Koch, veterinary investigators in Russia (Professor Gutman), the UK (John McFadyean), Denmark (Bernhard Bang), and the US (Leonard Pearson and Maz'yck Ravenel) were administering tuberculin to cattle as an *in vivo* diagnostic reagent (infection indicated by a rise in temperature within 24 hours) [2]. Clemens von Pirquet and Charles Mantoux later (circa 1907/1908) adapted and improved (e.g., subcutaneous to intradermal) this technology for application in the diagnosis of TB in humans, coincidently defining the principles of allergy and delayed type hypersensitivity. During the 1980s, an *in vitro* IFN-*γ* release assay was developed for the diagnosis of TB in cattle [3]; a modified version of this assay is now widely used in the diagnosis of both human and bovine TB. Together, these findings demonstrate the mutual benefit for cooperative veterinary and medical research. As stated by Emil von Behring in his Nobel Prize acceptance speech [4], **“**I need hardly add that the fight against cattle tuberculosis only marks a stage on the road which leads finally to the effective protection of human beings against the disease.” The current review highlights recent observations on immunity to bovine TB of relevance for understanding the disease, both in cattle and humans.

## 2. The Neonatal Calf as a Model for the Study of TB

### 2.1. *Mycobacterium bovis*

*Mycobacterium bovis*, a member of the *M. tuberculosis* complex, has a wide host range as compared to other species in this disease complex, is infectious to humans, and is the species most often isolated from tuberculous cattle. Prior to implementation of widescale pasteurization, it is estimated that 20–40% of TB cases in humans resulted from infection with *M. bovis* [5–7]. An explanation, not apparent at the time, suggests a difference in the capacity of *M. tuberculosis* and *M. bovis* to infect and cause disease in cattle. Genome comparisons show that *M. bovis* and *M. tuberculosis* evolved into two clades from a common prototypic ancestor some 40,000 years ago: clades defined by presence or absence of *M. tuberculosis *deletion 1 (TbD1) [8]. The data suggest that both clades arose in humans, with the TbD1^−^ clade 1 coevolving mainly in humans and the TbD1^+^ clade 2 coevolving in humans, ruminants, and other species. The difference in host range shows that evolution of *M. bovis* and *M. tuberculosis* has included development of a difference in virulence and the capacity to cause disease in different species. This difference may prove useful in comparative studies designed to elucidate the mechanisms of immunopathogenesis and development of vaccines. Approximately 90% of humans exposed to *M. tuberculosis* develop an immune response that controls but does not eliminate the pathogen. Immune control of this persistent (latent) stage of infection may persist for a lifetime or become dysregulated, allowing for disease progression. It is not clear whether a comparable proportion of humans infected with *M. bovis* develop an immune response that controls infection. Recent direct comparison of *M. bovis* and *M. tuberculosis* infection in cattle has demonstrated that *M. tuberculosis* is less virulent for cattle; however, the *M. tuberculosis* strain used for these studies was a laboratory-adapted strain (H_37 _R_v_) [9]. However, experimental transmission studies (conducted in the late 1800s by Theobald Smith (physician scientist) and veterinarians Austin Peters and Langdon Frothingham using calves experimentally inoculated with sputum from humans with tuberculosis), demonstrated that human bacilli possess a low virulence for cattle [10]. Other studies clearly demonstrated that nonlaboratory-adapted strains of *M. tuberculosis* were less virulent in cattle than those of *M. bovis* (reviewed by Whelan et al., 2010 [9]). Analysis of the difference in the immune response to the two pathogens may provide insight into the mechanisms used by both bacteria to circumvent protective immunity [11].

### 2.2. Aerosol Infection Model

Aerosol inoculation of *M. bovis* to calves results in a respiratory tract infection (i.e., lungs and lung-associated lymph nodes), severity is dose-dependent, and the disease closely mimics natural infection of cattle [12]. As related to human disease, studies with neonatal calves are particularly relevant as this is the primary target population for human vaccination and exposure to TB often occurs at a very young age. For calf vaccine studies, parameters to demonstrate efficacy include quantitative and qualitative mycobacterial culture, gross and microscopic disease scoring, radiographic morphometry of lung lesions, and disease-associated immune parameters. Opportunities afforded by use of the calf model include (1) large numbers of affordable age-, gender-, and breed-matched animals available throughout the year (nonhuman primates (NHPs) are seasonal breeders and are costly), (2) cattle are out-bred species, thus, experimental variance is similar to what is expected for NHP and humans, (3) size allows dose titration studies and full immunologic assessment via frequent collection of large volumes of blood which facilitates studies on immune response kinetics, (4) the nutritional status (e.g., vitamin deficiencies and protein malnutrition) can be manipulated to achieve similar levels of deficiency as may occur in target human populations in the developing world, (5) serves as an additional safety screen for evaluation of vaccines, adjuvants, or other administered biologics, and (6) feasibility of duration of immunity studies.

### 2.3. Parallel Testing of Vaccine Candidates in the Primate and Calf Model-mc^2^6030

A double deletion mutant of *M. tuberculosis* H_37 _R_v_ (i.e., ΔRD1-the primary attenuating mutation of BCG and ΔpanCD-deletion of pantothenate synthesis genes) was evaluated in parallel with BCG (Copenhagen strain) in both the calf model and adolescent Cynomologus monkeys (*Macaca fascicularis*). The *M. tuberculosis* ΔRD1 X panCD mutant (mc^2^6030) undergoes limited replication in mice and is safer than BCG in immunocompromised mice (i.e., SCID, IFN-*γ* receptor-deficient and CD4-deficient mice) [13]. Immunization with this mutant strain of H_37 _R_v_ induces prolonged protective immune responses that promote survival of both wild type and CD4-deficient mice against an aerosol challenge with virulent *M. tuberculosis*. Antibody depletion and adoptive transfer studies revealed that protection in CD4-deficient mice was mediated in the absence of CD4^+^, CD8^+^, *γδ* ^+^, and NK1.1^+^ T cells, thus, indicating a surprising capacity for protection to be elicited by CD4^−^CD8^−^ TCR−*αβ* ^+^ cells [14]. For second tier testing, mc^2^6030 was evaluated for efficacy in the newborn calf model and adolescent Cynomologus monkeys. In both calves and monkeys, the vaccine was ineffective [15, 16]; thus, in this instance, responses in mice were not predictive of efficacy in models using natural hosts of infection. For cattle, attenuated *M. tuberculosis* mutants may be less immunogenic as compared to those produced on a virulent *M. bovis* or BCG strain; thus, cattle may not be as useful as other models (e.g., monkeys) for the study of vaccine efficacy using *M. tuberculosis* mutants. Further studies are required to directly compare immunogenicity and virulence of *M. tuberculosis* versus *M. bovis* background mutants in cattle.

## 3. Bovine DCs and Macrophages

*The role of signal regulatory protein alpha-expressing *(SIRP*α* ^+^)* cells in the adaptive response to tuberculous mycobacteria via interactions with ESAT-6/CFP-10. *Multiple functions are proposed for the RD1 proteins ESAT-6 and CFP-10 [17, 18]. For instance, ESAT-6 interacts with biomembranes after dissociation from its putative CFP-10 chaperone within the acidic phagolysosome [19], thereby affording a “phagolysosome escape” mechanism for the pathogen. ESAT-6 deletion mutants of *M. tuberculosis* have reduced tissue invasiveness, likely due to loss of cytolytic activity [20]. In addition, use of the *M. marinum*/zebrafish granuloma model demonstrates that RD1 components are required for efficient recruitment of macrophages to granulomas “creating new bacterial growth niches” [21, 22]. RD-1 proteins, including ESAT-6/CFP-10, likely elicit more rapid granuloma formation offering a distinct growth advantage for the pathogen [22]. In addition to enhancing recruitment of cells susceptible to infection, the stable ESAT-6/CFP-10 complex binds to host cells [23]; thereby, modulating the host response favourably for the pathogen via down-regulation of host cell killing mechanisms and immune cell activation [24]. 

A specific receptor (TLR2) for ESAT-6 has been identified using mouse monocyte/macrophage cell lines [25]. Studies with leukocytes obtained from cattle have also demonstrated a specific interaction of the ESAT-6/CFP-10 complex with CD172a (SIRP*α*)-expressing cells [26]. Stimulation of peripheral blood mononuclear cell cultures from *M. bovis*-infected calves with ESAT-6/CFP-10 results in the specific expansion of SIRP*α* ^+^ cells, with binding of the fusion protein bound to the surface of SIRP*α* ^+^ cells [26]. SIRP*α*-CD47 interactions are essential for efficient migration of DCs to skin [27] and secondary lymphoid organs [28]. Thus, ESAT-6/CFP-10-induced expansion of SIRP*α*-expressing cells may favour migration of DC's/macrophages to infection sites, thereby, promoting efficient granuloma formation and early dissemination of *M. tuberculosis* complex mycobacteria [22]. With the bovine TB model, intradermal injection of rESAT-6:CFP-10 elicits granuloma formation with infiltration of numerous T cells, SIRP*α* ^+^ cells, and CD14^+^ cells in *M. bovis*-infected calves, further supporting a role for ESAT-6/CFP-10 in the recruitment of naïve cells to sites of granuloma formation [26]. A unique aspect of the cellular infiltrates at rESAT-6:CFP-10 injection sites in cattle is the presence of numerous multinucleated giant cells. Multinucleated giant cell formation is mediated, in part, by SIRP*α* (also termed macrophage fusion receptor). Cell surface expression of SIRP*α* is strongly and transiently induced upon giant cell formation. As opposed to phagocytosis, SIRP*α*-CD47 interactions provide “self recognition” signals that prevent killing of internalized (i.e., fused) cells. Thus, cattle may serve as a useful model for the molecular characterization of multinucleate giant cell formation within tuberculous granulomas. Additionally, comparative studies examining ESAT-6/CFP-10 interactions with SIRP*α* and TLR2 in mouse, human, and bovine tissues are needed to determine if host factors affect ESAT-6/CFP-10 specificity.

## 4. T Cell Subsets and Effector Mechanisms

### 4.1. Cell-Mediated Immunity (CMI)

Ongoing studies have shown the adaptive immune system of cattle is quite similar to the human system [29]. Importantly, comparisons at the genomic level indicate that genes encoding cytokines known to play a role in regulating immune responses in humans are present in cattle, including cytokines not found in mice (e.g., IL-26). Cell-mediated immunity is essential for protection against bovine and human TB and there appears to be significant similarity in the primary mechanisms of antimycobacterial CMI between humans (as reviewed in [30]) and cattle (as reviewed in [31]). Similarities include roles of T cell subsets (e.g., CD4^+^, CD8^+^, and *γδ* TCR^+^), protective function and cellular sources of IFN*γ* and cytotoxic granule proteins; the reduction of mycobacterial numbers within macrophages by cytotoxic T cells and NK cells, the relative levels of antigen specific Th1 and Th2 cytokines, and expression of memory markers by antigen specific T cells [32–42]. Widely utilized for TB diagnosis, antigen-specific release of IFN-*γ* is clearly an important function of the CMI response to TB in cattle and humans [43, 44]. In contrast to rodents, human and bovine immunity to TB appears to be less reliant on antigen-specific IFN-*γ* activation of macrophages [45, 46] and may employ cytotoxic immune cells in a more active role. 

Protective immunity to TB in cattle, as in human and other animal models of TB, correlates to the induction of Type 1 T cell cytokines following antigen specific stimulation [45, 47]. The balance of cytokines (IFN-*γ*, IL-4) elicited by mycobacterial antigens in bovine T cells, however, is more similar to cytokine profiles observed following immunization of humans than of mice. Relative levels of IFN-*γ* and IL-4 expressed by lymph node T cells correlate to tissue pathology and bacterial load in vaccination/challenge studies of bovine TB [15, 48]. In BCG-vaccinated neonatal calves, antigen-specific expression of IL-2 and IFN-*γ* by peripheral blood leukocytes correlated with clinical protection following challenge with virulent *M. bovis * [48]. A potential role for the Th2/Th17 cytokine IL-21 in protection against mycobacterial disease was recently identified for the first time in a calf model of TB [35]. IL-21 is a member of the common gamma chain family of cytokines (IL-2, IL-7, IL-15) and is secreted primarily by CD4^+^ T cells [49]. Following vaccination with BCG, CD4^+^ T cells from immunized cattle expressed IL-21 upon *in vitro* stimulation with PPD [35]. Expression of IL-21 in these studies correlated with cytotoxic activity and effector molecule expression by antigen-stimulated CD4^+^ T cells and occurred late in the antigen-specific response, similar to perforin. The role of IL-21 and other key regulatory factors for maintenance and induction of IFN-*γ* (IL-12, IL-18, IL-23, and IL-27) and NK cell function in protective immunity to TB is an important avenue of investigation in the efforts to develop a vaccine for humans and cattle.

### 4.2. *γδ* T Cells in Bovine Tuberculosis

**γδ* T* cells may form a key component linking innate and adaptive responses to *M. bovis* infection in cattle given their active release of IFN-*γ* and relatively high prevalence in the blood of young calves as compared to adults [50]. There are two phenotypically distinct subsets of *γδ* T cells in cattle, workshop cluster 1 (WC1)^+^ CD2^−^ and WC1^−^ CD2^+^ *γδ* T cells. Unique to *Artiodactyla*, WC1 is a member of the scavenger cysteine rich gene family that includes CD5 and CD6 [51]. Differences in abundance, tissue distribution, patterns of circulation, and TCR gene usage suggest that the two major *γδ* T cell subsets (WC1^+^ and WC1^−^) play different roles in host defense [52–55]. Orthologues of WC1 have only been identified in pigs and camelids [51, 56–59] but there is no known orthologue of WC1 in primates. Isoforms of WC1 are encoded by a cluster of thirteen genes distributed between two loci in cattle [51, 56–58] and studies have demonstrated that multiple gene products from the two loci are coexpressed forming two essentially mutually exclusive populations with an apparent dichotomy in function [60, 61]. These two subsets—referred to as WC1.1^+^ and WC1.2^+^—are defined by differential staining with specific monoclonal antibodies. Functional studies have demonstrated that the subset expressing WC1.1 is a major source of IFN-*γ* following antigenic stimulation [55] and is likely an early source of perforin and granulysin. The relative proportions of WC1.1^+^and WC1.2^+^cells change with age, with a predominance of WC1.1^+^ *γδ* T cells in young cattle [62]. In addition, we showed that WC1^+^ *γδ* T cells from young cattle had a greater capacity for IFN*γ* secretion, compared to WC1^+^ *γδ* T cells from adult cattle, and that this was due to a higher number of WC1.1^+^ cells in the young calves [63]. 

*γδ* T cells respond to mycobacterial antigens including crude and defined antigens, heat shock proteins, and other nonproteinaceous components which may be expressed relatively early in infection with *M. bovis* or other mycobacterial species [64–66]. The effector contribution of the WC1^+^ *γδ* T cells to *M. bovis* immunity in cattle is not fully elucidated but early roles postinfection and postvaccination are indicated from a number of studies in cattle and in mice. Vaccination of calves with BCG induced an early increase in circulating WC1^+^ *γδ* T cells which was associated with an increase in the secretion of antigen-specific IFN-*γ* [67]. We also showed that intranasal administration of BCG induced significant increases in WC1.1^+^ T cells in the lungs of immunised calves and that these cells clustered with DC in tissues [50]. In *M. bovis*-infected cattle, WC1^+^ *γδ* T cells were detected within lesions early in the course of infection [68, 69]. Concurrently, numbers of circulating WC1^+^ *γδ* T cells decrease shortly after infection, followed by a rapid increase [66]. *In vivo* depletion of WC1^+^ *γδ* T cells from cattle prior to infection did not alter the course of disease [70] but, rather, significantly influenced the immune bias of the antigen-specific response. These data suggest that WC1^+^ cells may be involved in directing immune bias through IFN-*γ* secretion, as the WC1^+^ *γδ* T cell-depleted cattle had increased IL-4 expression and altered immunoglobulin isotype profile as compared to nondepleted, *M. bovis*-infected controls [70]. Studies in mice have also revealed roles for *γδ* T cells in immune responses to *Mycobacteria spp.*. The WC1-bearing *γδ* T -cell population was shown to have an essential role in regulating inflammation in both the liver and the spleen of *M. bovis*-infected SCID-bovine heterochimeric (mouse-bovine) chimeras [37]. A similar regulatory effect of *γδ* T lymphocytes in inflammatory responses induced by *M. tuberculosis* was also reported to influence bacterial survival within tissues [71]. The early presence of *γδ* T cells in tuberculous lesions likely promotes containment of *M. bovis* via cytokine (e.g., IL-12 and IFN-*γ*), granulysin, and chemokine release stimulating macrophage activation and T cell recruitment [70, 72, 73]. However, recent studies in the SCID-bovine heterochimeric mouse model have shown that *γδ* T cells are not a primary source of chemokines in response to various agonists but may influence other cells types (including macrophages) in the production of chemokines and granuloma formation [74]. Treatment of mice with an anti-WC1 monoclonal antibody resulted in an apparent loss of control of the inflammatory response confirming important roles for WC1^+^ *γδ* T cells in vivo in regulation of the immune response. Similar findings were obtained with mycobacterial infection of *γδ* TCR knockout mice [75, 76]. Interestingly other studies have also shown that it is the bovine WC1.1^+^ and WC1.2^+^ *γδ* T cells which act as T regulatory cells, not CD4^+^CD25^+^FoxP3^+^ cells as has been observed in other species [77]. The available data suggest that bovine WC1^+^ *γδ* T cells have multiple roles and can be both regulatory and stimulatory through the expression of cytokines. Their exact roles in *M. bovis* immunity remain to be fully revealed.

### 4.3. Cytotoxic Lymphocytes (CTLs) and Biomarkers for Effector Mechanisms in Bovine TB

Human and murine CD4^+^, CD8^+^, and *γδ* T cells exhibit CTL activity against mycobacterial-infected targets, indicating a role in immunity to TB [78–81]. Production of granulysin by CTL reduces intracellular mycobacteria and appears to require perforin to gain access to the interior of the infected cell. Bovine T cells express a homologue of human granulysin, a potent antimicrobial protein stored with perforin in cytotoxic granules [34]. Memory CD4^+^ T cells (CD45R0^+^) from BCG-vaccinated animals are efficient at reducing colony forming units of BCG in infected macrophages following antigen specific stimulation [35]. This antimycobacterial activity correlates with expression of perforin, granulysin, and IFN-*γ* by the same memory subset. Expression of the bovine granulysin gene can be induced in CD4^+^, CD8^+^, and *γδ* T cells resulting in antimycobacterial activity similar to human granulysin [34, 35]. Using laser capture microdissection, granulysin mRNA was detected in the lymphocytic cuff of a forming granuloma, simultaneous with *M. bovis* DNA, in an experimentally challenged calf [34]. Granulysin and perforin gene expression are upregulated in peripheral blood CD4^+^ and CD8^+^ T cells in both BCG- and *M. bovis* ΔRD1-vaccinated calves (protected) as compared with nonvaccinated (not protected) calves [82], demonstrating the potential of these biomarkers as correlates of protection for prioritizing vaccine candidates. To date, a murine and guinea pig orthologue of granulysin has not been identified, precluding studies of the full repertoire of lytic granule proteins in rodent models of TB. Further studies of protective biomarkers in peripheral blood and granulomas of vaccinated and experimentally infected cattle have significant potential for testing strategies for augmenting immunity by vaccination.

Bovine NK cells have been identified with a mAb specific for CD335 [83, 84] and numbers of NK cells in peripheral blood were found to be highest in young calves [83, 85]. The population is comprised of CD2^+^ and CD2^−^ subsets expressing combinations of killer immunoglobulin-like receptors (KIRs), leukocyte-receptor complex (LRC) CD94/NKG2C (inhibitory) and CD94/NKG2A (activating), NKG2D, and lectin-like receptors Ly49, CD69, NKP-R1, and KLRJ (reviewed in [86]). Initial studies suggest that NK cells play a significant role in the innate response to mycobacterial pathogens [33, 87–89]. They are an initial source of IFN-*γ*, IL-17, and IL-22 that play a role in the inflammatory response to intracellular pathogens, including *M. tuberculosis*, and a source of perforin and granulysin. Bovine neonatal NK cells were shown to be a major source of IFN*γ* through interactions with BCG-infected DCs and this may be a pivotal early influence *in vivo* [88]. 

NKT cells have not been identified in cattle. Analysis of the CD1 family of proteins involved in antigen presentation to NK and NKT cells suggests NKT cells may not be present in cattle or that receptor usage differs markedly from that in humans and mice. The CD1 family in cattle is comprised of genes encoding CD1a, multiple CD1b molecules, and CD1e. An orthologue of the human CD1c gene is not present in the bovine genome. Both identified CD1d genes are pseudogenes supporting the possibility that NKT cells may be absent [90].

## 5. Immunological Parameters as Correlates of Protection versus Indicators of Disease

### 5.1. T Cell Central Memory (TcM) Immune Responses

Costly and protracted efficacy studies using various and often multiple animal models are currently used to evaluate human TB vaccine candidates [91]. Vaccine-elicited immune parameters (i.e., correlates of protection) are very much needed to prioritize the multitude of candidates. Several vaccine studies with cattle have demonstrated that TcM responses [92] negatively correlate with mycobacterial burden [93] and TB-associated pathology [94]; thus, TcM responses are positive correlates of protection. With the TcM assay, short-term T cell lines are generated via stimulation of peripheral blood mononuclear cells with specific antigens including Ag85A/TB10.4 and *M. bovis* PPD. Early effector T cell responses wane over time and memory cells are maintained via addition of IL-2 and fresh medium. On the final day of culture (~13d), cells are moved to plates containing autologous antigen presenting cells and antigen for elicitation of TcM responses as measured by IFN-*γ* ELISPOT. With two independent vaccine efficacy trials [93, 94], protected calves had greater TcM responses as compared to nonprotected calves. As with TcM responses, IL-17 responses (as measured by real time RT-PCR) also correlated with protection [94]. Recent findings indicate that IL-21 and IL-22 may also be good candidates for further evaluation (*Davis and Waters, unpublished observations*). These data demonstrate the potential for defining a protective signature elicited by vaccination to prioritize candidates for efficacy testing within calves.

### 5.2. Immune Activation as a Positive Indicator of Disease and Negative Indicator of Vaccine Efficacy

Positive prognostic indicators (i.e., as measured after challenge) for vaccine efficacy include reduced antigen-specific IFN-*γ*, iNOS, IL-4, and MIP1-*α* (CCL3) responses, reduced expansion of CD4^+^ cells in culture, and a diminished activation profile (i.e, ↓ expression of CD25 and CD44 and ↑ expression of CD62L) on T cells with antigen-stimulated cultures [15]. In particular, reduced responses to ESAT-6/CFP-10 upon experimental challenge are positive prognostic indicators for vaccine efficacy [44, 93, 95] whereas robust or increasing cellular immune responses (with the exception of IL-17 [94]) to ESAT-6/CFP-10 are generally a negative prognostic indicator of vaccine efficacy. These findings are consistent with the diagnostic capacity for ESAT-6/CFP-10 as antigens in cellular immune assays. Prognostic indicators offer ante-mortem monitoring techniques for vaccine efficacy studies.

### 5.3. Association of Antibody Responses to Antigen Burden

Antibody responses generally correlate to mycobacterial-elicited pathology [96] in accordance with the belief that *Mycobacteria spp.* induce antibody primarily late in the course of infection. To further evaluate the correlation of antibody responses to disease expression, calves were inoculated with *M. bovis*, *M. kansasii*, or *M. tuberculosis* and immune responses evaluated [9, 11]. Disease expression ranged from mycobacterial colonization with associated pathology (*M. bovis*), colonization without pathology (*M. tuberculosis*), to no colonization or pathology (*M. kansasii*). Antibody responses were associated with antigen burden; cellular responses (i.e., to PPD) correlated with infection but not necessarily with pathology or bacterial burden; exposure to mycobacterial antigens (in this case, injection of PPD for skin test) boosted antibody responses in presensitized animals. Thus, evaluation of antibody response to mycobacterial infections may be useful for correlation to antigen burden and prior mycobacterial sensitization. Further studies are warranted.

## 6. Global Strategies for Discovery: Gene Expression Profiling

One argument for use of rodent models in human tuberculosis research was the widespread availability of reagents to detect cytokines, chemokines, and for differentiation of immune cells. The advent of functional genomics, proteomics, and completion of the bovine genome sequence have dramatically improved our ability to study immunopathogenesis of TB in cattle. In addition, there has been a steady increase in numbers of antibody reagents either prepared directly against bovine proteins or validated for cross-reactivity against bovine orthologues. To date, functional genomics studies of *M. bovis* infection in cattle have been concentrated in two main areas: (1) *in vitro* studies aimed at defining changes in macrophage gene expression profiles following infection with various *M. bovis* isolates and (2) *ex-vivo* studies to define a “gene expression signature” that could be used to detect *M. bovis*-infected cattle. Many of these studies have relied upon early renditions of cDNA microarrays focused on bovine genes encoding proteins known to be important in immunity [97–100].

Wedlock et al. compared gene expression patterns in primary bovine alveolar macrophages infected with a virulent *M. bovis* strain and its attenuated isogenic counterpart [101]. This study employed a cDNA microarray containing over 20,000 bovine-expressed sequence tags (ESTs) as well as amplicons representing various bovine and cervine cytokines [102]. While virulent and attenuated *M. bovis* isolates grew at comparable rates in the alveolar macrophages, initial analyses suggested that as many as 45% of the ESTs were differentially expressed between the two infection groups [101]. Of the 20 most differentially expressed genes, IL-8, monocyte chemotactant protein (MCP)-1 (CCL2), epithelial cell inflammatory protein-1, Gro*α* (CXCL1), CDC-like kinase 3, and fibrinogen-like protein-2 were prominent. Wedlock et al. concluded that alveolar macrophages infected with virulent *M. bovis* adopted a much more proinflammatory gene expression profile than similar cells infected with the attenuated isogenic strain [101]. Another study using microarray technology found that lower levels of chemokines were expressed by *M. bovis-*infected alveolar macrophages than *M. tuberculosis*-infected cells, highlighted by the authors as a potential mechanism by which *M. bovis *can circumvent activation of the host chemotactic response and evade killing [103]. Of note, there appears to be species differences in the response of macrophages to *M. bovis* as human monocytes and monocyte-derived macrophages (MDM cells) infected with *M. bovis* do not produce significant IL-8 [104]. In addition, human cells infected with virulent *M. tuberculosis* produce large amounts of IL-10 [104], which was not observed in *M. bovis*-infected alveolar macrophages. However, as acknowledged by the authors, it remains a possibility that these discrepancies are due to differences between MDM cells and primary alveolar macrophages rather than actual species differences. Experiments to refine these observations have not been reported to date.

Meade et al. conducted a series of studies aimed at defining the gene expression profiles of bovine peripheral blood mononuclear cells (PBMCs) from *M. bovis*-infected cattle following stimulation with antigens of *M. bovis* (PPD-B) in vitro [99]. Samples were collected throughout a 24 hour time course of stimulation. Comparisons were made primarily between PPD-B-stimulated and -unstimulated PBMC mRNA profiles. This study utilized a bovine-specific cDNA microarray (BOTL-4) designed primarily for immune studies in cattle and containing over 1300 ESTs and amplicons spotted in triplicate [105, 106]. Statistical analysis revealed that of the >1300 genes present on the BOTL-4 microarray, 224 (~17%) were differentially expressed in PPD-B-stimulated PBMCs when compared to unstimulated PBMCs from the same chronically infected animals [99]. Major ontological classes of genes that showed significant differential expression included those encoding proteins involved in metabolism, cell communication, response to biotic stimulus, death, and development. Molecular functions most affected by stimulation were catalytic activity, protein binding, and nucleic acid binding. During the 24-hour stimulation time course, the authors observed a cyclical gene expression pattern with larger numbers of transcripts differentially expressed at 3-hour and 12-hour post stimulation relative to 6-hour and 24-hour time points. Although this could be related to cyclical receptor signalling, the fact that very few transcripts were commonly affected throughout the time course suggests that it is due to activation of early response transcripts followed by a lag (possibly due to transcription/translation of early response transcripts) followed by a secondary wave of gene expression. Alternatively, this pattern could be due to immediate response to mycobacterial antigens through, for example, Toll-like receptor signalling followed by a secondary stimulation after uptake, processing, and antigen presentation. The authors suggested that this early response pattern might represent a signature of *M. bovis* infection [99]. 

In a series of subsequent studies, Meade et al. pursued the idea that a unique and rapid gene expression pattern in PBMCs could be used to reliably detect *M. bovis*-infected cattle [107, 108]. Biomarker discovery using genomics and proteomics has been applied to infections with other pathogens in several host species, including humans and cattle [109–112]. Meade et al. demonstrated that total leukocytes from late-stage *M. bovis*-infected cattle contained more lymphocytes (72%) than similar samples from control healthy cattle (43%) and that healthy controls contained more neutrophils (40%) than cells from late-stage *M. bovis*-infected cattle (14%) [113]. Given such differences, one would expect that total leukocyte gene expression profiles from late-stage *M. bovis*-infected cattle would be different than those from healthy controls. Indeed, of the >1,300 genes represented on the BOTL-5 microarray, 378 (27%) showed significant differential expression (*P* <  .05) between total leukocytes from healthy and infected cattle. Importantly, the suppression of innate immune gene expression detected in chronically infected animals [113] could be one mechanism by which *M. bovis* infection persists [31], and current diagnostics fail to identify all infected animals. Of importance, application of a hierarchical clustering algorithm identified a subset of 15 genes whose combined expression pattern appeared to be indicative of infection status [99]. It may also be the case that the early and transient profile of differential gene expression detected in these studies, could shed light on the mechanisms and kinetics of the shift in the immune system toward a nonprotective, antibody-mediated response associated with progression to chronic disease [31, 47]. It is also possible that expression patterns of this gene subset could be used to diagnose *M. bovis* infection in cattle. Unfortunately, this was not rigorously tested using a blinded set of samples in the present study. However a recent review from this group suggests that work in this area is on-going [114]. It waits to be seen if changes in expression of such a gene subset are specific to *M. bovis* infection or are a common response to bacterial invasion.

In order to address the issue of an *M. bovis* specific gene expression pattern, Meade et al. performed another time course study using *M. bovis* antigen stimulation to elicit changes in gene expression that would not necessarily be common with any other bacterial infection [107, 108]. This study revealed a subset of 18 genes that showed opposite expression changes (up- or downregulated) in cells from healthy control cattle and *M. bovis*-infected cattle. In keeping with previous results and relevant literature, the nature of the differentially expressed genes suggested a significant role for Toll-like receptor signalling in response to *M. bovis* antigens [107, 108, 113, 114]. This is postulated to have significant consequences for the development of disease in humans [115], and the same may hold true in cattle.

While there may be some species differences between the response of humans and cattle to mycobacterial infections, as discussed elsewhere in this article, there are fewer significant differences between cattle and humans than between mice and humans. This can have significant advantages for translational understanding of the mechanisms of pathogenesis [116]. Detailed pan-genomic transcriptomic analyses will also aid the understanding and potential therapeutic targeting of *M. bovis* infections in other natural infection models, including wildlife species that pose threats as reservoirs of infection [117–119]. Significant progress has been made in defining a gene expression signature that may be diagnostic for *M. bovis* infection in cattle. In many cases, eradication of *M. bovis* is difficult because currently used diagnostic tests do not detect all infected cattle. Development of an improved test, based on genomic or proteomic biomarkers, would be a great improvement. Particular focus also needs to be paid to the development of early stage indicators of infection in order to minimize the losses and risks associated with advanced or chronic infection. Evidence for differential cytokine gene expression, found to be associated with pathology in *M. bovis* infected cattle [120], and a pre-existing gene expression profile in infected animals holds promise for such a resolution [108, 113]. In fact a recent study has made considerable progress in this area using comparative proteomic analysis to identify biomarkers of subclinical (latent) *M. bovis *infection [121]. Serum levels from experimentally infected animals showed marked increases of alpha-1-microglobulin/bikunin precursor (AMBP) protein, alpha-1-acid glycoprotein, alpha-1-microglobulin and fetulin proteins in *M. bovis *infected animals, which were absent from animals challenged with other closely related mycobacteria. As yet, genomic and proteomic tests have not been subjected to rigorous field testing, presumably because funding sources for a large blinded study have not been available. In addition, transfer of genomics information from *M. bovis* infected cattle to *M. tuberculosis* (and *M. bovis*) infections in humans has not been forthcoming. However, functional genomics has added new dimensions to our understanding of the bovine immune response to *M. bovis* infection, and the advent of new technologies including next-generation sequencing holds significant potential for the development of novel intervention strategies against this recalcitrant disease.

## 7. Summary

The advent of genomic resources for cattle has dramatically improved our ability to use this species in studies of immunity to a variety of diseases, including zoonotic infections such as *M. bovis*. Although the mouse model has proven useful in comparative studies of tuberculosis, recent advances in the characterization of the immune system of cattle now afford an opportunity to gain further insight into the mechanisms of immunopathogenesis utilizing a natural host/pathogen relationship. It is clear from recent investigations that the Th1/Th2 paradigm must be expanded to include the newly identified CD4 and CD8 T cells subsets that comprise the effector and regulatory subsets responding to mycobacterial antigens during different stages of infection. Further studies are needed to determine the relative contribution of Treg and Th17 subsets in the response of cattle to *M. bovis* infection/vaccination. Efforts to elucidate differences between the immune response profile of individuals with latent infection and patients with progressive disease suggest that latency is associated with maintenance of subsets of CD4 T cells [117]. The calf TB model affords a unique opportunity for evaluation of neonatal immune responses to vaccination/infection. Promising TB vaccines may also be evaluated in the calf (safety and efficacy) prior to testing in costly NHPs (thereby prioritizing candidates), and as presented in this review, important mechanisms of immunity may be uncovered by the use of the calf for the study of TB.
